# The Distribution of Obesity Phenotypes in HIV-Infected African Population

**DOI:** 10.3390/nu8060299

**Published:** 2016-06-02

**Authors:** Kim Anh Nguyen, Nasheeta Peer, Anniza de Villiers, Barbara Mukasa, Tandi E. Matsha, Edward J. Mills, Andre Pascal Kengne

**Affiliations:** 1 Non-Communicable Diseases Research Unit, South African Medical Research Council, Cape Town 7505, South Africa; Kim.Nguyen@mrc.ac.za (K.A.N.); nasheeta.peer@mrc.ac.za (N.P.); Anniza.DeVilliers@mrc.ac.za (A.d.V.); 2 Department of Medicine, University of Cape Town, Cape Town 7935, South Africa; 3 United Nations Population Fund (UNFPA), Mildmay Uganda PO Box 24985, Lweza, Uganda; barbara.mukasa@mildmay.or.ug; 4 Department of Biomedical Sciences, Faculty of Health and Wellness Science, Cape Peninsula University of Technology, Cape Town 7535, South Africa; matshat@cput.ac.za; 5 Global Evaluation Science, Vancouver, BC V6H 3X4, Canada; emills@redwoodoutcomes.com

**Keywords:** obesity phenotype, metabolic abnormalities, HIV infection

## Abstract

The distribution of body size phenotypes in people with human immunodeficiency virus (HIV) infection has yet to be characterized. We assessed the distribution of body size phenotypes overall, and according to antiretroviral therapy (ART), diagnosed duration of the infection and CD4 count in a sample of HIV infected people recruited across primary care facilities in the Western Cape Province, South Africa. Adults aged ≥ 18 years were consecutively recruited using random sampling procedures, and their cardio-metabolic profile were assessed during March 2014 and February 2015. They were classified across body mass index (BMI) categories as normal-weight (BMI < 25 kg/m^2^), overweight (25 ≤ BMI < 30 kg/m^2^), and obese (BMI ≥ 30 kg/m^2^), and further classified according to their metabolic status as “metabolically healthy” *vs.* “metabolically abnormal” if they had less than two *vs.* two or more of the following abnormalities: high blood glucose, raised blood pressure, raised triglycerides, and low HDL-cholesterol. Their cross-classification gave the following six phenotypes: normal-weight metabolically healthy (NWMH), normal-weight metabolically abnormal (NWMA), overweight metabolically healthy (OvMH), overweight metabolically abnormal (OvMA), obese metabolically healthy (OMH), and obese metabolically abnormal (OMA). Among the 748 participants included (median age 38 years (25th–75th percentiles: 32–44)), 79% were women. The median diagnosed duration of HIV was five years; the median CD4 count was 392 cells/mm^3^ and most participants were on ART. The overall distribution of body size phenotypes was the following: 31.7% (NWMH), 11.7% (NWMA), 13.4% (OvMH), 9.5% (OvMA), 18.6% (OMH), and 15.1% (OMA). The distribution of metabolic phenotypes across BMI levels did not differ significantly in men *vs.* women (*p =* 0.062), in participants below *vs.* those at or above median diagnosed duration of HIV infection (*p* = 0.897), in participants below *vs.* those at or above median CD4 count (*p* = 0.447), and by ART regimens (*p* = 0.205). In this relatively young sample of HIV-infected individuals, metabolically abnormal phenotypes are frequent across BMI categories. This highlights the importance of general measures targeting an overall improvement in cardiometabolic risk profile across the spectrum of BMI distribution in all adults with HIV.

## 1. Introduction

People living with HIV infection constitute a sizable proportion of the world population and the number is increasing [1]. The advent and uptake of antiretroviral therapy (ART) has turned HIV infection from a highly fatal infectious disease into a chronic manageable condition [2]. Consequently, the lifespan of HIV infected patients receiving ART is now close to that of the general population [3]. This has led to a rise in chronic and age-related conditions such as cardio-metabolic disorders in HIV-infected people [4,5], that is contributing substantially to the overall morbidity and mortality in this population [6,7].

A major contributor to cardio-metabolic diseases is the global obesity epidemic with 52% (1.9 millions) of the worldwide adult population being either overweight or obese in 2014 [8]. Obesity contributes to cardio-metabolic abnormalities by impairing metabolic functions that promote dyslipidemia, insulin resistance, as well as chronic inflammation [9]. Consequently, concepts such as “metabolically healthy” and “metabolically abnormal” have been used to characterize individuals across the distribution of body mass index (BMI) as a function of the underlying burden of metabolic abnormalities [10].

The changes in body fat distribution associated with HIV infection are well-described [11]. The advanced stage of untreated HIV infection is associated with changes in body fat content and distribution, which are partially and perhaps non-optimally restored following treatment with ART [12]. ART extends the lifespan of HIV-infected people by reducing the viral load with a subsequent strengthening of the immune system; notably, it does not eliminate the HIV infection. Hence, chronic inflammation persists and there is incomplete restoration of the immune system [13]. Additionally, various metabolic abnormalities are associated with HIV infection and its related treatments [14,15]. These include dyslipidemia, insulin resistance, and abnormal blood pressure levels, [13], which contribute to cardiovascular diseases (CVDs) and type 2 diabetes mellitus (T2DM) [13,16].

While obesity and HIV infection have been extensively researched separately, there is a dearth of data on the distribution of obesity phenotypes in HIV-infected people [17]. Therefore, in the current study, we assessed the distribution of obesity phenotypes, and the effects if any, of ART and other major distinctive characteristics of HIV infection, in a representative sample of people with HIV recruited across primary healthcare facilities in the Western Cape Province, South Africa.

## 2. Materials and Methods

### 2.1. Study Design and Sampling Procedure

A cross-sectional survey was conducted from March 2014 to February 2015 in a random sample of HIV-infected adults aged 18 years and older being treated at public healthcare facilities across the Western Cape Province in South Africa. Permission to conduct the survey was obtained from Health Research Office of the Western Cape Department of Health, and the relevant healthcare facilities.

The healthcare facilities considered for this study needed to provide ART to at least 325 HIV-infected patients per month to ensure adequate recruitment within a reasonable period. Thus, the sample frame comprised a total of 62 healthcare facilities with 42 across Cape Town and 20 in the surrounding rural municipalities. Of these, 17 facilities, including four rural, were randomly selected for inclusion in this study. At each participating healthcare facility, 15–60 patients were randomly sampled.

The study was approved by the South African Medical Research Council Ethics Committee and conducted in accordance with the principles of the Declaration of Helsinki.

### 2.2. Data Collection

A team of trained clinicians, nurses, and field workers collected data by administering questionnaires, clinical measurements, and biochemical analyses. Data were captured on electronic case report forms, which were available on personal digital assistants (PDAs), with built-in checks for quality control. Data were encrypted at the point of collection and sent via mobile connection to a dedicated server, from which it was further checked, downloaded, and stored for future use. The interviews and the physical assessments were conducted on the day of recruitment while the blood specimens were drawn the following day after the participant had fasted overnight.

#### 2.2.1. Interviews

Socio-demographic data and medical history were obtained using a structured interviewer-administered questionnaire adapted from the World Health Organization’s STEPwise approach to Surveillance (STEPS) tool. Self-reported data included duration of being diagnosed HIV infection and CD4 counts, whereas information on HIV treatment was obtained by capturing medications brought to by the participants.

#### 2.2.2. Physical Examination

Anthropometric parameters including height, weight, and waist circumference (WC) were measured using standardized techniques. Height was measured to the nearest millimeter using a Leicester Height Scale (Seca, Liverpool, UK) with the participant barefoot and in the upright position. Weight was measured to the nearest gram using A&D Personal Scale (Model UC-321, Toshima-Ku, Tokyo, Japan) with the participant in light clothes, and without shoes. WC, recorded to the nearest millimeter, was taken at the level of umbilicus. After the participant was seated in a resting position for at least five minutes, blood pressure (BP) was measured in mmHg on the right arm, using a digital automatic BP monitor (Omron, M6 Comfort, Hoofddorp, The Netherland); three measurements were taken three minutes apart.

#### 2.2.3. Laboratory Measurements

Biochemical parameters were analyzed at an ISO 15189 accredited pathology laboratory (PathCare, Reference Laboratory, Cape Town, South Africa) which had no access to participants’ clinical information. All analyses were performed on venous blood samples collected after an overnight fast of at least eight hours. Serum cholesterol and triglycerides were measured by enzymatic colorimetric methods; ultrasensitive C-reactive protein was read; plasma glucose was measured by hexokinase method; all implemented using a Beckman Coulter AU 500 spectrophotometer. Insulin concentrations were measured by the Chemiluminesecence Immunoassay method while HbA1c level was determined using high-performance liquid chromatography technique. The homeostatic model assessment of insulin resistance (HOMA-IR) was calculated as the product of insulin (mIU/L) and glucose (mmol/L) by 22.5 [18].

### 2.3. Definitions

#### 2.3.1. Socio-Demographic Characteristics

Education level was distinguished into primary education and secondary education or above. Smoking status was categorized as never-smoker, past-smoker (stopped smoking during the past 12 months), and current-smoker. Alcohol intake behavior was classified as non-heavy drinker (consumed <5 standard alcoholic drinks for men and < 4 standard alcoholic drinks for women in a row during the past 30 days), and heavy-drinker (consumed ≥5 standard alcoholic drinks for men and ≥4 standard alcoholic drinks for women in a row during the past 30 days). A standard alcoholic drink was corresponding to one can (340 mL) of beer, one glass (125 mL) wine, or one shot (25 mL) of spirits. Duration of diagnosed HIV infection was the time since being diagnosed with HIV. ARTs were categorized as first line ART, second line ART, and other regimens [19].

#### 2.3.2. Body Size Phenotype

Body mass index (BMI) was calculated as weight (kg)/height × height (m^2^). BMI was used to classify participants into three categories: normal weight (BMI < 25 kg/m^2^), overweight (BMI ≥ 25 kg/m^2^ and BMI < 30 kg/m^2^) and obese (BMI ≥ 30 kg/m^2^). There is no consensus on the definition and number of cardio-metabolic abnormalities to use when characterizing obesity phenotypes [20]. In the current study, we considered the following four abnormalities: (1) elevated BP determined using the average of the second and third BP measurements (systolic BP ≥ 130 mmHg or diastolic BP ≥ 85 mmHg or known hypertension on treatment); (2) high triglycerides (≥1.69 mmol/L); (3) low high-density lipoprotein cholesterol (HDL-C ≤ 1.0 mmol/L in men; ≤1.3 mmol/L in women); (4) high blood glucose (fasting plasma glucose (FPG) ≥ 5.6 mmol/L or known diabetes mellitus). In secondary analysis, in a subset of participants with data available on insulin levels, we included insulin resistance as a fifth metabolic abnormality, by classifying as insulin resistant all participants with HOMA-IR above the data specific 90th percentile. Considering the lack of consensus on waist circumference (or waist-to-hip ratio) threshold to define abdominal obesity in African populations, the criteria of abdominal obesity which has been included in about 30% of studies on obesity phenotype [20] was not included in our panel of metabolic abnormalities.

Participants were then classified for metabolic status as “metabolically healthy” if they had none or one metabolic abnormality, and as “metabolically abnormal” if they had two or more metabolic abnormalities. Cross-classification of participants by BMI and metabolic status led to the following six phenotypes: (1) normal weight and metabolically healthy (NWMH); (2) normal weight and metabolically abnormal (NWMA); (3) overweight and metabolically healthy (OvMH); (4) overweight and metabolically abnormal (OvMA); (5) obese and metabolically healthy (OMH); and (6) obese and metabolically abnormal (OMA).

### 2.4. Statistical Analysis

Participants’ characteristics are summarized as means (standard deviation, SD) and medians (25th to 75th percentiles) for continuous variables, and as count (percentages) for categorical variables. Comparison of baseline characteristics across BMI categories, by metabolic status, and by HIV-related characteristics were done using chi-square tests, fisher-exact test, *t*-tests or Kruskal-wallis tests for non-parametric data or Analysis of Variance test (ANOVA) where appropriate. The linear trends across BMI categories overall and by metabolic status were examined using the Cochrane-Armitage trend tests and Brown-Forsythe Levene procedures. The two-way interactions between BMI categories and metabolic status (B × M), and metabolic status and gender (M × G) were tested using linear and logistic regression models, by incorporating in the same model the main effects of the variables of interest as well as their interaction term.

To assess the association between each continuous metabolic trait and BMI categories, multinomial logistic regressions models (age and sex adjusted) were used to derive the odds ratio (OR) and 95% confidence interval for a unit higher level of each metabolic trait in relation with overweight and obesity risk, always using normal weight are reference category. The McFadden’s *R*^2^ [21] was then used as a measure of the overall performance of models containing age, gender, and each metabolic trait of interest. A two-side *p*-value < 0.05 indicates a statistical significance. All analyses were performed using the R statistical software version 3.0.3 (the R Foundation for Statistical Computing Platform, Vienna, Austria. For a *z*-value of 1.96 (corresponding to a 95% confidence interval), and an effective sample size of 748 participants, our study had a margin of error of 0.05% to detect a prevalence of normal weight metabolically abnormal phenotype of 1% in the total sample. The acceptable margin of error is 5%, indicating that our study was well-powered for the overall and subgroup analyses.

## 3. Results

### 3.1. Socio-Demographic Characteristics

Of the 831 participants who were interviewed and clinically assessed, 754 (91%) returned for biochemical measurements. Blood samples from six participants were inadequate for analyses resulting in 748 (99%) participants being included in the study. This comprised 591 women and 157 men who had complete data on the variables of interest.

The median age (25th–75th percentiles) was 38 (32–44) years overall, 41 (35–47) years in men, and 37 (31–43) years in women (*p* < 0.001). As shown in Table 1, most participants (84.9%) had secondary education or higher with a lower prevalence in men (75.8%) than in women (87.3%) (*p* < 0.001). About half of the participants (54.6%) were employed with similar rates in men and women (49.7% *vs.* 55.9%, *p* = 0.162). Current smoking was more prevalent in men than in women (58.8% *vs.* 16.1%, *p* < 0.001) but heavy alcohol consumption was similar (34.1% *vs.* 34.1%, *p* = 0.975).

### 3.2. Profile of HIV Infection

The median duration of diagnosed HIV infection was five years (25th–75th percentiles: 2–9) with no difference by gender (*p* = 0.223). The median CD4 count was 392 cells/mm^3^ (25th–75th percentiles: 240–604) with higher levels in women than in men (410 cells/mm^3^ *vs.* 272 cells/mm^3^, *p* = 0.002). Most participants were receiving ART (93.4%) with the majority on first line ART (63.9%), while 11.8% received second line ART and 17.4% were on other ART regimens. Interestingly, there were significant differences in the distribution by gender (*p* = 0.005).

### 3.3. Profile of Cardio-Metabolic Abnormalities

The mean BMI was 26.3 kg/m^2^ overall with significantly lower levels in men compared with women (21.4 kg/m^2^ *vs.* 28.3 kg/m^2^, *p* < 0.001). Overall, 43.4% of participants had normal BMI levels while 22.9% were overweight and 33.7% obese with significant differences by gender (*p* < 0.001). Women compared to men had larger WCs (90 cm *vs.* 79 cm, *p* < 0.001), higher HOMA-IR indices (1.49 *vs.* 0.94, *p* < 0.001), and total cholesterol levels (4.4 *vs.* 4.2 mmol/L, *p* = 0.009). However, they had lower levels of triglycerides (0.97 *vs.* 1.12 mmol/L, *p* = 0.023), fasting glucose (4.9 *vs.* 5.1 mmol/L, *p* = 0.010) and systolic BP (115 *vs.* 124 mmHg, *p* < 0.001). Furthermore, HDL-cholesterol levels (1.29 *vs.* 1.2 mmol/L, *p* = 0.010) and prevalent treated hypertension (16.8% *vs.* 7.0%, *p* = 0.002) were higher in women than men. Diastolic BP, hs-CRP, as well as prevalent treated diabetes were similar in both genders (all *p* ≥ 0.129) (Table 1).

### 3.4. Distribution of Body Size Phenotypes

**Figure 1:**
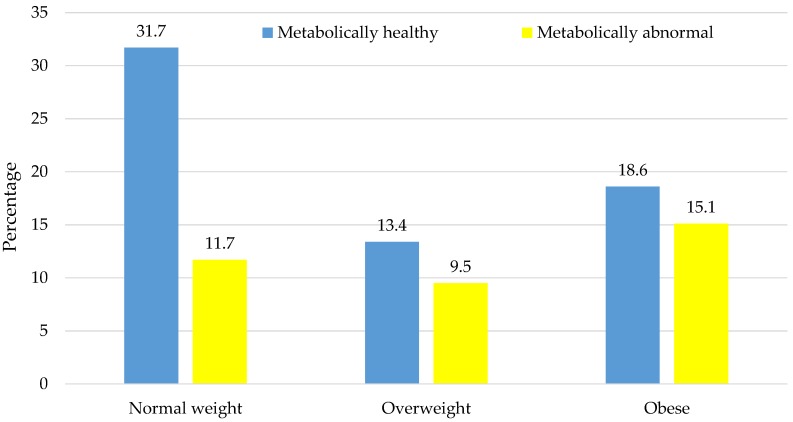
Distribution of metabolic phenotypes across body mass index categories. Each vertical bar represents the proportion of participants in the total sample with the corresponding combination of body size (normal-weight, overweight, or obese) and metabolic phenotype (healthy or abnormal). The accompanying proportions are shown at the tip of each bar.

The proportion of ≥2 metabolic abnormalities across normal-weight (27.1%), overweight (41.5%), and obese (44.8%) categories increased significantly in a linear trend (*p*-trend = 0.001). The distribution of body size phenotypes in the overall sample was 31.7% (NWMH), 11.7% (NWMA), 13.4% (OvMH), 9.5% (OvMA), 18.6% (OMH), and 15.1% (OMA), Figure 1.

**Figure 2:**
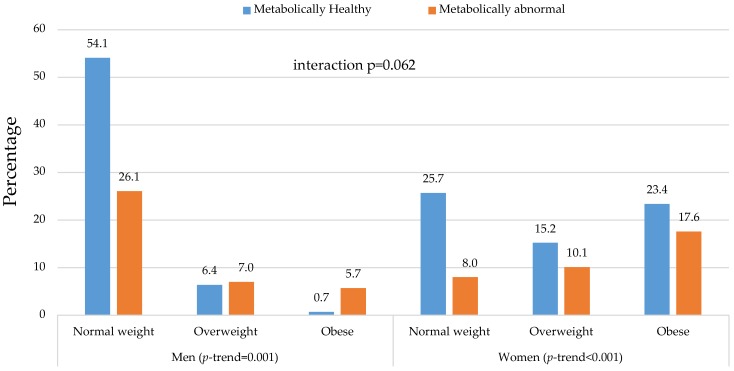
Distribution of metabolic phenotypes across body mass index categories in men and women. Each vertical bar represents the proportion of participants in the total gender-specific sub-sample with the corresponding combination of body size (normal-weight, overweight, or obese) and metabolic phenotype (healthy or abnormal). The accompanying gender-specific proportions are shown at the tip of each bar. The *p*-value for the interaction by gender in the distribution are shown, together with the *p*-value for le linear trend (*p*-trend) in the distribution of metabolic phenotypes across body mass index categories, seperately in men and women.

In men, the majority (54.1%) were NWMH, just over a quarter (26.1%) were NWMA, while few fell into the other categories: 6.4% (OvMH), 7% (OvMA), 0.7% (OMH), and 5.7% (OMA). In contrast, the distribution in women was as follows: 25.7% (NWMH), 8% (NWMA), 15.2% (OvMH), 10.1% (OvMA), 23.4% (OMH), and 17.6% (OMA) (Figure 2). There was no statistically significant interaction by gender in the distribution of body size phenotypes (*p*-interaction = 0.062).

**Figure 3:**
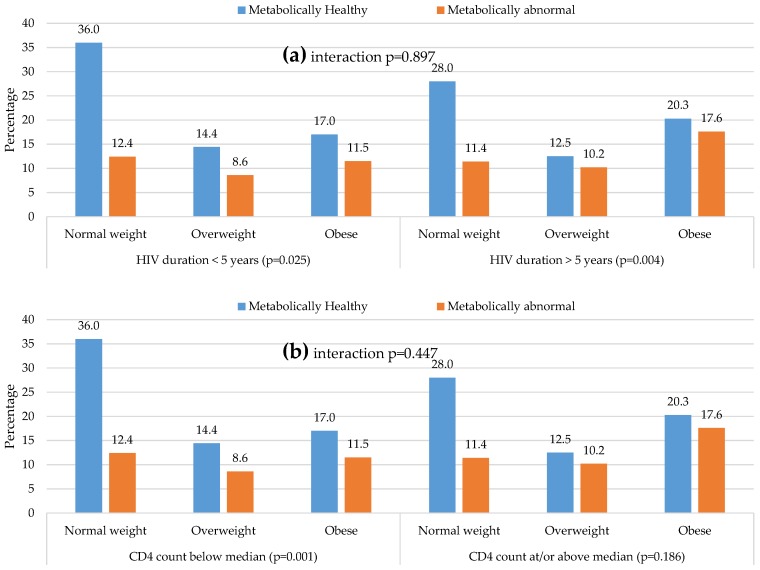

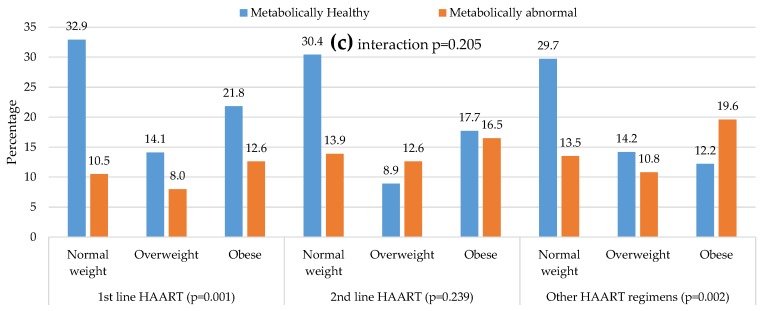
Distribution of body size phenotypes by major HIV predictive characteristics: (**a**) Distribution of metabolic phenotype across body mass index categories in participants below, and those at or above the median of diagnosed duration of HIV infection; (**b**) Distribution of metabolic phenotype across body mass index categories in participants below, and those at or above the median CD4 count; (**c**) Distribution of metabolic phenotype across body mass index categories in participants on different antiretroviral treatment regimens. For each figure panel, the *p*-values for the interactionb (interaction *p*) in the distribution across complementary subgroups are, together with the *p*-value for linear trend in the distribution of metabolic phenotype across body mass index categories within each subgroup (*p*-value attached to the name of the subgroup). Each vertical bar represents the proportion of participants in the subgroup specific sample with the corresponding combination of body size (normal-weight, overweight, or obese) and metabolic phenotype (healthy or abnormal). The accompanying proportions are shown at the tip of each bar.

The distribution of metabolic phenotypes across BMI categories was not significantly different by longer or shorter duration of diagnosed HIV infection (median five years, *p*-interaction = 0.897) (Figure 3a), higher or lower CD4 count (median 392 cells/mm^3^, *p*-interaction = 0.447) (Figure 3b) or across the three ART regimens (*p*-interaction = 0.205) (Figure 3c). The proportion of ≥2 metabolic abnormalities tended to increase significantly and linearly across BMI categories within most of the latter subgroups except for lower CD4 count and the second line ART regimen.

### 3.5. Distribution of Metabolic Phenotypes within and Across BMI Categories

Within BMI categories, in addition to the expected differences in the levels of cardio-metabolic risk factors, participants with metabolically abnormal phenotypes tended to be older (all *p* ≤ 0.001) and unemployed, although differences were significant only among normal weight and overweight (all *p* ≤ 0.002), but not among obese participants (both *p* ≥ 0.215). Furthermore, metabolically abnormal obese participants were likely to be men (8.0% *vs.* 0.7%, *p* = 0.006) and included fewer participants on first line ART (*p* = 0.009).

Age and level of education across BMI categories increased linearly overall (both *p* ≤ 0.012), driven by a significant linear trend in metabolically healthy participants (both *p* ≤ 0.005) but not in the metabolically abnormal (both *p* ≥ 0.396), with however no evidence of statistical interaction (both *p*-interaction ≥ 0.065). The proportion of men who were current smokers decreased linearly overall across increasing BMI categories (*p* < 0.001 for linear trend), and within both metabolic phenotype groups (*p*-trend = 0.001), with no evidence of statistical interaction (both interactions *p* > 0.759), Table 2.

Median WC, HOMA-IR, and HDL-C levels across BMI categories increased significantly overall (*p*-trend ≤ 0.001) and within the metabolic phenotype groups (all *p*-trend ≤ 0.024), without evidence of statistical interaction (all *p*-interaction ≥ 0.560). Fasting glucose, triglycerides, and prevalence of hypertension also increase across increasing BMI categories, but only on the total cohort (*p*-trend ≤ 0.038). Interaction analyses found BMI categories and metabolic status interacted to affect the distribution of fasting glucose (*p*-interaction = 0.044) whereas metabolic status interacted with gender to influence triglycerides distributions across BMI categories (*p*-interaction = 0.002).

**Figure 4:**
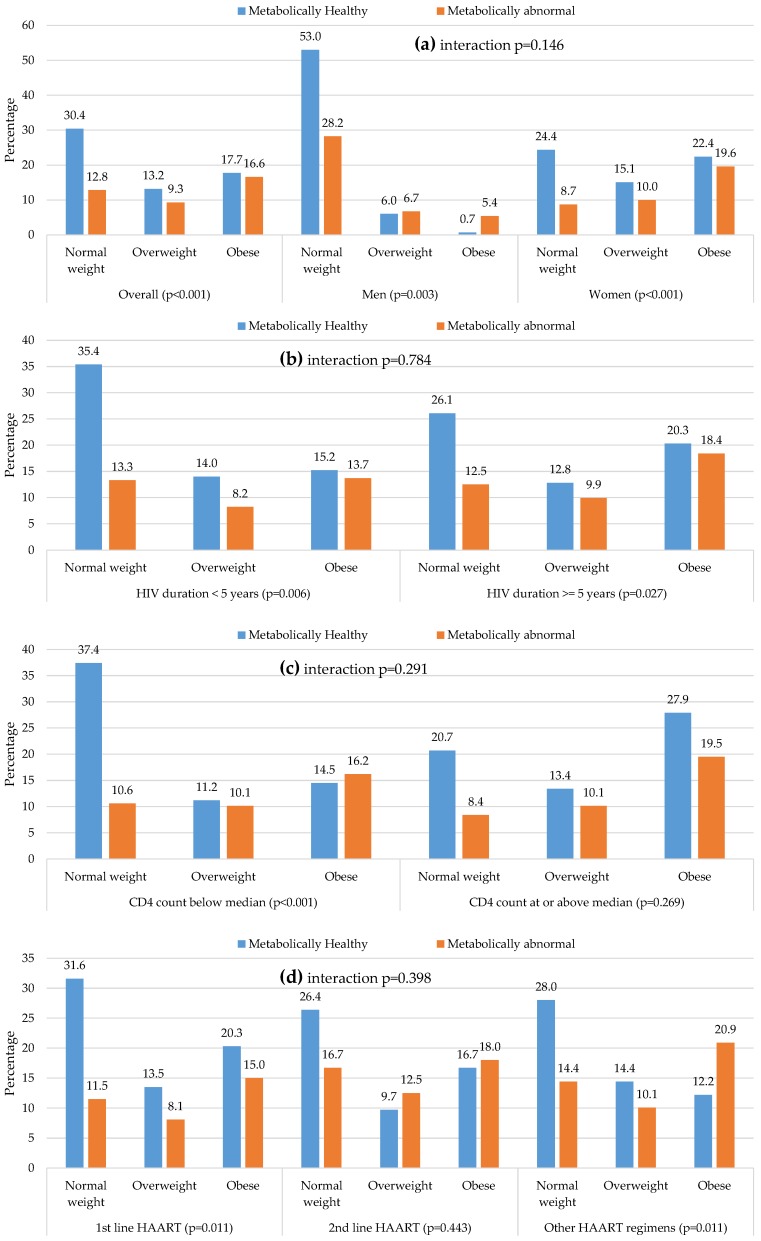
Distribution of metabolic phenotypes across body mass index by major characteristics: (**a**) Overall and in men and women; (**b**) in participants below, and those at or above the median of diagnosed duration of HIV infection; (**c**) in participants below and those at or above the median CD4 count; (**d**) in participants on different antiretroviral treatment regimens. Metabollically abnormal phenotype is based on the presence of any two of the following five abnormalities: elevated blood pressure or known hypertension; high triglycerides; low HDL-cholesterol, high blood glucose, or known diabetes; insulin resistance. For each figure panel, the *p*-values for the interaction (interaction *p*) in the distribution across complementary subgroups are, together with the *p*-value for linear trend in the distribution of metabolic phenotype across body mass index categories within each subgroup (*p*-value attached to the name of the subgroup). Each vertical bar represents the proportion of participants in the subgroup specific sample with the corresponding combination of body size (normal-weight, overweight, or obese) and metabolic phenotype (healthy or abnormal). The accompanying proportions are shown at the tip of each bar.

Moreover, when further analyses in the subgroup of participants with data on insulin level (*n* = 711) that included insulin resistance (HOMA-IR in 90th) as a fifth metabolic abnormality. The prevalence ≥2 risk factors was found to increase slightly across BMI categories: NWMA (12.8%), OvMA (9.3%), and OMA (16.6%), but the patterns within and across subgroups were mostly similar (Figure 4).

### 3.6. Prediction of Body Mass Index Categories by the Continuous Metabolic Traits

In multinomial logistic regression models, mutually adjusted for each other and using normal weight as a reference, male sex was associated with 81% (95% confidence interval: 68%–89%) lower odds of overweight and 94% (89%–97%) lower odds of obesity; while each year of older age was associated with 2% (0%–4%) higher odds of overweight and a non-significant 1% (−1% to 4%) higher odds of obesity. The McFadden *R*^2^ for the overall performance of this basic model was 0.081. In the presence of age and sex, all metabolic trait with the exception of systolic blood pressure (for both overweight and obesity) and fasting plasma glucose (for overweight only) were significantly associated with odds of overweight and obesity (Table 3). The direction of the effect with increasing metabolic traits levels was always positive, except for HDL-cholesterol where increasing levels were associated with decreasing odds of overweight and obesity. The highest *R*^2^ for the overall performance of resulting models was recorded for the model containing HOMA-IR (*R*^2^ = 0.183); and ranged from 0.083 (for the model containing systolic blood pressure) to 0.103 (for the model containing either triglycerides or HDL-cholesterol), Table 3.

## 4. Discussion

Although there have been rigorous reports on fat distribution and obesity in individuals with HIV infection, to our knowledge, this is one of the first studies to document the distribution of cardio-metabolic abnormalities in relation with body size in an HIV-infected population. Metabolically abnormal phenotypes, defined as the presence of ≥2 cardio-metabolic risk factors, were high across all BMI categories. Even in normal weight participants, over a quarter (27.1%) had metabolically abnormal phenotypes with this rising to 41.5% and 44.8% in the overweight and the obese, respectively. This suggests the likely influence of multiple factors in the development of cardio-metabolic abnormalities in this population. The high prevalence in normal weight HIV-infected individuals suggests the possible contribution of HIV-related factors and underscores the need to examine for cardio-metabolic abnormalities even in the absence of overweight and obesity.

In contrast, the much higher prevalence demonstrated with increasing adiposity may be attributable to the greater role of this conventional risk factor in the development of cardio-metabolic abnormalities in the HIV-infected population. This highlights the fine balance that needs to be maintained between ensuring adequate nutrition and optimal weight in the HIV-infected while simultaneously monitoring and guarding against excess weight gain. Thus, there is a need for holistic management of these co-morbidities that may indirectly be associated with HIV infection.

The prevalence of overweight and obesity, at 13.4% and 6.4% in men and 25.4% and 40.9% in women in this study approximated the adiposity distribution reported in South African National Health and Nutrition Survey (SANHANES-1) [22]. The overweight and obesity rates in the SANHANES-1 were 20.1% (95% CI: 13.7–26.4) and 11.6% (95% CI: 7.5–15.7) in men and 26.4% (95% CI: 21.7–31.0) and 44.8% (95% CI: 38.8–50.8) in women, respectively. Similar findings have been reported in the United States where obesity levels in HIV-infected men (19%) and women (42%) were comparable to the general population (men: 24.7%, women: 37%) [17]. It is thus important to assess adiposity in HIV-infected individuals and to implement appropriate management strategies for weight reduction, similar to general populations. Notably, that the distribution of overweight and obesity in this study mirrors that of the general population in the country is testimony of the successful implementation of ART strategies in this community.

Although the prevalence of the metabolically abnormal phenotypes by the BMI categories described in this study were significant at 11.7% (NWMA), 9.5% (OvMA), and 15.1% (OMA), a substantial proportion of participants were obese but metabolically healthy (18.6%). This agrees with the 6%–75% estimate of OMH in general populations globally and is likely because BMI is a proxy marker of cardiovascular risk. BMI measures general fat distribution and not visceral adipose tissue specifically, which is linked closely to insulin resistance and cardio-metabolic abnormalities. A better proxy for visceral adipose tissue is WC and, unsurprisingly, within BMI categories, participants with compared to without metabolic abnormalities had greater WC and elevated HOMA-IR index, a proxy measure of insulin resistance. Similar findings have also been reported in other studies [20,23,24,25]. Recent studies in Caucasians that have included insulin resistance among abnormal metabolic traits, have reported high prevalence of metabolically abnormal phenotypes both in obese and non-obese people [26,27].

The distribution of body size phenotypes in our study is comparable with results of local studies conducted in the general population in South Africa [24,28]. Our previous community-based study in mixed-ancestry adults in Cape Town applying the same definition criteria found OMH and NWMA to be present respectively 16.5% and 5% of the sample [24]. Furthermore, among normal-weight participants (17.1% of the sample), 29.1% were classified as metabolically abnormal, while among obese participants (53.7% of the sample), 30.8% were classified as metabolically healthy [24]. In another study in 103 normal-weight and 122 obese premenopausal urban black South African women, Jennings and co-workers found that 22% of the normal-weight participants were metabolically abnormal (defined by the presence of insulin resistance), while 38% of obese women were metabolically healthy (*i.e.*, did not have insulin resistance) [28]. There are no recent reports available from Africa for comparison of the metabolically abnormal phenotypes that include insulin resistance. Nevertheless, data from a study conducted almost two decades ago in Cameroon revealed a much lower prevalence of metabolic abnormalities with 1.4% (NWMA), 1.6% (OvMA), and 1.7% (OMA) [23]. This highlights the epidemiological transition under way in Sub-Saharan Africa with the majority of this study’s participants having normal weight (61%) in the year 1994, unlike more recent reports, and the expected lower prevalence of cardio-metabolic risk factors compared with the present study [23]. Interestingly, there was no significant difference in the distribution of metabolic abnormalities across BMI groups by duration of diagnosed HIV infection, CD4 count levels or ART regimens. There have been diverse reports on the effects of HIV-specific factors on body fat de-arrangement, dyslipidemia, hyperglycemia, and metabolic syndrome. However, results from a recent systematic review and meta-analysis indicated that HIV-related characteristics had minor, if any, influence on the presence of metabolic syndrome [29]. Nevertheless, longitudinal studies are ideally required to pronounce on the absence or presence, if any, of a relationship between specific HIV-related factors and the development of cardio-metabolic abnormalities by body size phenotype.

The relationship of gender, smoking status, and alcohol consumption on the distribution of body size phenotypes remains inconclusive in studies conducted in general populations [10,30,31]. The findings of this study accorded with reports that showed little or no effect of smoking status and alcohol use on the distribution of body size phenotypes [26]. However, a few studies showed a higher prevalence of OMH in women than in men [26,32].

### Limitations and Strengths

The cross-sectional design of this study precludes inferences of causal associations between the variables of interest and the development of cardio-metabolic abnormalities. The inclusion of an HIV-uninfected and HIV-infected ART-naïve comparative groups would have strengthened our analyses. Seeing that this was a clinic-based study limits its generalizability since it did not include HIV-infected individuals not attending healthcare facilities. However, these limitations are inevitable because the present project is part of a broad intervention study, which aims to explore the utilization of HIV-care infrastructure as a gateway to detect, manage, and control non-communicable diseases in HIV-infected populations in Africa. The relatively fewer men compared to women in the study, characteristic of epidemiological studies in the country, might overestimate the prevalence of obesity phenotypes. In the absence of detailed information on dietary habits/food consumption and data on ethnicity, we could not explore possible effects of lifestyle factors and ethnicity on the distribution of body size phenotypes among the participants. Nevertheless, differences in MHO prevalence according to ethnicity have been reported, although this recent meta-analysis did not include any studies based on African cohorts [33]. There are reports indicating that overall dietary intake was not associated with healthy obesity in both Europeans and African Americans [27,34]. In addition to lifestyle and ethnicity, data was not available on the pharmacological compounds included in the ART regimens of the participants as well as the duration of treatment with those compounds, precluding detailed analyses by potency of pharmacological compounds and duration of treatments.

Nonetheless, the inclusion of participants from 17 healthcare facilities, including both urban and rural sites strengthens the representativeness in terms of the characteristics assessed. Furthermore, this study is among the first to describe the high prevalence of metabolically abnormal phenotypes across BMI categories in a relatively young HIV-infected population. The study findings underscore the need for further research, particularly longitudinal studies, to understand the development of cardio-metabolic abnormalities in the local HIV-infected population and the differential role played by conventional risk factors as opposed to HIV-related influences.

## 5. Conclusions

The high prevalence of metabolically abnormal phenotypes across all BMI categories, notably in a relatively young HIV-infected population, highlights the importance of holistic management in HIV-infected individuals. Ideally, cardio-metabolic assessments/screenings should be done at baseline and at regular intervals thereafter, particularly in high-risk groups. Furthermore, considering the high prevalence of overweight and obesity in the HIV-infected, lifestyle measures for weight reduction need to be encouraged. This is a captive audience who present regularly to healthcare facilities and the opportunity should be used to raise greater awareness on cardiovascular disease prevention. Such a strategy, targeting all HIV-infected patients, may contribute to a general improvement in cardiovascular health across the spectrum of BMI distribution. If proven successful, it may possibly have wider applicability in the general population.

## Figures and Tables

**Table 1:** Characteristics of the HIV/AIDS patients (*n* (%), or median (25th–75th percentiles)).

Characteristics	Overall, (*n =* 748)	Men, (*n =* 157)	Women, (*n* = 591)	*p*
Age, year	38 (32–44)	41 (35–47)	37 (31–43)	<0.001
Education level, *n* (%)				<0.001
Primary	113/746 (15.1)	38/157 (24.2)	75/589 (12.7)	
Secondary and above	633/746 (84.9)	119/157 (75.8)	514/589 (87.3)	
Employed, *n* (%)	408/747 (54.6)	78/157 (49.7)	330/590 (55.9)	0.162
Smoking habit, *n* (%)				<0.001
Never smoke	461/718 (64.7)	34/156 (22.2)	427/562 (76.4)	
Current smoker	187/718 (25.3)	93/156 (58.8)	90/562 (16.1)	
Past smoker	70/718 (13.3)	29/156 (45.3)	42/562 (9.0)	
Heavy drinker, *n* (%)	64/187 (34.2)	22/64 (34.4)	42/123 (34.1)	0.975
HIV duration, years	5 (2–9)	4 (2–7)	5 (2.5–9)	<0.001
CD4, cells/mm^3^	392(240–604)	272(193–448)	410(253–627)	0.001
ART treatment, *n* (%)				0.005
Non-ART	46/699 (6.6)	7/149 (4.7)	39/550 (7.1)	
first line	426/699 (60.9)	78/149 (52.3)	348/550 (63.3)	
second line	79/699 (11.3)	17/149 (11.4)	62/550 (11.3)	
Others	148/699 (21.2)	47/149 (31.5)	101/550 (18.3)	
Body mass index (kg/m^2^)				
Median (P25–P75)	26.3 (22.1–32)	21.4 (19.8–22.4)	28.3 (23.8–28.9)	<0.001
<25, *n* (%)	325 (43.4)	126 (80.3)	199 (33.7)	
25.0–29.9, *n* (%)	171 (22.9)	21 (13.4)	150 (25.4)	
≥30, *n* (%)	252 (33.7)	10 (6.4)	242 (40.9)	
Waist circumference, cm	88 (77.5–98)	78.9 (73.9–88.3)	90 (79.5–100.8)	<0.001
Systolic BP, mmHg	117 (107–129.5)	123.5 (114.5–140)	115 (105.8–127)	<0.001
Diastolic BP, mmHg	82 (75–90.5)	83 (76–94)	81.5 (74.8–89.8)	0.129
Total cholesterol, mmol/L	4.3 (3.7–5.1)	4.2 (3.5–5.0)	4.4 (3.8–5.1)	0.009
HDL-cholesterol, mmol/L	1.27 (1.03–1.5)	1.2 (1.0–1.5)	1.29 (1.08–1.52)	0.010
LDL-cholesterol, mmol/L	2.5 (2.0–3.1)	2.3 (1.7–3.0)	2.5 (2.0–3.1)	0.012
Triglycerides, mmol/L	1.0 (0.74–1.34)	1.12 (0.75–1.27)	0.97 (0.74–1.28)	0.023
Fasting glucose, mmol/L	5.0 (4.6–5.4)	5.1 (4.8–5.5)	4.9 (4.6–5.4)	0.010
HOMA-IR	1.36 (0.84–2.24)	0.94 (0.53–1.64)	1.49 (0.93–2.37)	<0.001
C-reactive protein, mg/L	5.6 (2.4–12)	5.0 (2.1–16.2)	5.6 (2.4–14.2)	0.728
Treated hypertension, *n* (%)	110 (14.7)	11 (7)	99 (16.8)	0.002
Treated diabetes, *n* (%)	28 (3.7)	8 (5.1)	20 (3.4)	0.432

ART, antiretroviral; BP, blood pressure; HDL, high density lipoprotein; HIV, human immunodeficiency virus; AIDS, acquired immunodeficiency syndrome; HOMA-IR, homeostatic model assessment of insulin resistance; LDL, low density lipoprotein.

**Table 2:** Characteristics of participants across body mass index (BMI) categories and metabolic status [*n* (%), or median (25th–75th percentiles)].

BMI Categories	Normal Weight (*n =* 325)			Overweight (*n =* 171)			Obese (*n =* 252)			*p*-Trend			*p*-Interaction	
Metabolic Status	Healthy	Abnormal	*p*	Healthy	Abnormal	*p*	Healthy	Abnormal	*p*	Overall	Healthy	Abnormal	B × M	M × G
Prevalence, *n* (%)	237 (31.7)	88 (11.7)		100 (13.4)	71 (9.5)		139 (18.6)	113 (15.1)		<0.001	-	-	-	-
Men, *n* (%)	85 (35.9)	41 (46.6)	0.078	10 (10.0)	11 (15.5)	0.281	1 (0.7)	9 (8.0)	0.006	<0.001	<0.001	<0.001	0.759	-
Age, years	36 (30–44)	42 (34-49)	<0.001	36 (31–42)	43 (36–47.5)	<0.001	37 (31.5–41)	39 (34–47)	0.001	0.002	<0.001	0.396	0.065	0.34
≥7 school-years, *n* (%)	190/236 (80.5)	71/88 (80.7)	0.972	89/100 (89.0)	58/70 (82.9)	0.249	128/139 (92.1)	97/113 (85.8)	0.111	0.012	0.005	0.617	0.532	0.067
Unemployed, *n* (%)	84/236 (35.6)	48/88 (54.5)	0.002	38/100 (38.0)	47/71 (66.2)	<0.001	64/139 (46.0)	58/113 (51.3)	0.404	0.081	0.131	0.132	0.056	0.061
Smoking habit, *n* (%)			0.523			0.327			0.144	<0.001	<0.001	<0.001	0.925	0.238
Never	105/230 (45.7)	33/85 (38.8)		78/96 (81.3)	49/68 (72.0)		111/132 (84.1)	85/107 (79.4)						
Current smoker	99/230 (43.0)	40/85 (47.1)		12/96 (12.5)	11/68 (16.2)		15/132 (11.4)	10/107 (9.3)						
Past smokers	26/230 (11.3)	12/85 (14.1)		6/96 (6.2)	8/68 (11.8)		6/132 (4.5)	12/107 (11.2)						
Heavy drinkers, *n* (%)	26/74 (35.1)	8/27 (29.6)	0.643	7/25 (28.0)	8/19 (42.1)	0.356	7/22 (31.8)	8/20 (40.0)	0.748	0.973	0.801	0.638	0.518	0.766
HIV diagnosed duration, years	4 (2–7.8)	5 (2–8)	0.577	4.3 (2–8)	6 (2–9)	0.149	5 (3–10)	6 (4–10)	0.334	0.413	0.435	0.436	0.820	>0.999
Median CD4 count, /mm^3^	311 (172–473)	350 (232–544)	0.288	433 (187–630)	395 (252–626)	0.494	452 (297–677)	434 (267–699)	0.627	0.335	0.213	0.627	0.77	0.430
Antiretroviral regimens, *n* (%)			0.448			0.201			0.009	0.947	0.386	0.963	0.363	0.179
First line	140/208 (67.3)	45/76 (59.2)		60/88 (68.3)	34/60 (56.6)		93/125 (74.4)	54/96 (56.2)						
Second line	24/208 (11.5)	11/76 (14.5)		7/88 (8.0)	10/60 (16.7)		14/125 (11.2)	13/96 (13.5)						
Others	44/208 (21.2)	20/76 (26.3)		21/88 (23.7)	16/60 (26.7)		18/125 (14.4)	29/96 (30.2)						
Waist circumference, cm	77 (72–80)	78 (72–86)	0.016	89 (85–92)	93 (86–95)	0.005	101 (95–108)	104 (99–111)	0.005	<0.001	<0.001	0.016	0.560	0.780
Systolic blood pressure, mmHg	114 (105–125)	128 (116–145)	<0.001	112 (104–124)	125 (117–140)	<0.001	113 (106–119)	124 (114–138)	<0.001	0.230	0.136	0.560	0.620	0.610
Diastolic blood pressure, mmHg	78 (72–85)	88 (81–92)	<0.001	81 (73–85)	88 (82–97)	<0.001	81 (75–85)	88 (80–96)	<0.001	0.954	0.771	0.819	0.230	0.510
Fasting glucose, mmol/L	4.9 (4.6–5.2)	5.3 (4.8–6.3)	<0.001	4.9 (4.6–5.2)	5.2 (4.7–5.7)	0.001	4.9 (4.6–5.2)	5.6 (5.0–6.4)	<0.001	0.010	0.583	0.049	0.044	0.510
Median HOMA-IR	0.85 (0.57–1.27)	1.16 (0.82–1.79)	<0.001	1.31 (0.931.81)	1.76 (1.05–2.49)	0.003	1.9 (1.33–2.44)	2.52 (1.54–4.67)	<0.001	<0.001	0.001	0.006	0.630	0.290
Diabetes ^a^, *n* (%)	2/227 (0.9)	21/87 (24.1)	<0.001	1/96 (1.0)	10/68 (14.7)	0.001	2/128 (1.6)	27/110 (24.6)	<0.001	0.077	0.839	0.252	0.148	0.752
Hypertension ^b^, *n* (%)	52 (21.9)	50 (56.8)	<0.001	20 (20.0)	43 (60.6)	<0.001	35 (25.2)	70 (62.0)	<0.001	0.038	0.615	0.757	0.841	0.107
Triglycerides, mmol/L	0.9 (0.7–1.2)	1.2 (1.0–1.9)	<0.001	0.9 (0.7–1.2)	1.2 (1.0–1.9)	<0.001	0.9 (0.7–1.2)	1.4 (1.0–1.9)	<0.001	0.033	0.647	0.472	0.720	0.002
HDL-cholesterol, mmol/L	1.4 (1.1–1.7)	1.2 (0.9–1.3)	<0.001	1.4 (1.2–1.7)	1.1 (1.0–1.2)	<0.001	1.4 (1.2–1.6)	1.1 (1.0–1.2)	<0.001	0.001	0.021	0.024	0.790	0.640
LDL-cholesterol, mmol/L	2.2 (1.8–2.9)	2.5 (1.9–3.1)	0.181	2.4 (2.0–3.0)	2.5 (2.0–3.3)	0.423	2.6 (2.2–3.1)	2.8 (2.3–3.4)	0.019	0.867	0.769	0.713	0.910	0.330
Total cholesterol, mmol/l	4.2 (3.6–5.0)	4.2 (3.5–4.9)	0.483	4.3 (3.7–5.1)	4.2 (3.6–4.9)	0.665	4.5 (3.9–5.1)	4.5 (4.0–5.2)	0.208	0.126	0.350	0.438	>0.999	0.710
C-reactive protein, mg/L	4.2 (1.5–12.1)	5.2 (2.5–16.1)	0.102	4.4 (2.3–10.4)	4.4 (2.0–8.5)	0.959	7.8 (3.5–15.8)	8.0 (3.8–16.6)	0.590	0.803	0.523	0.236	0.770	0.130

^a^ Diabetes as FPG ≥ 7.0 mmol/L or on treatment; ^b^ hypertension as blood pressure (BP) ≥ 140/90 mmHg or on treatment.

**Table 3:** Odds ratios (OR) and 95% confidence intervals (95% CI) from multinomial age and sex adjusted multinomial logistic regression models, showing the association of metabolic traits with body mass index categories.

Predictors	Normal Weight	Overweight		Obese		*R*^2^
	Reference	OR (95% CI)	*p*-Value	OR (95% CI)	*p*-Value	
Age, per year	1.00	1.02 (1.00–1.04)	0.093	1.01 (0.99–1.04)	0.195	0.081
Sex, men	1.00	0.19 (0.11–0.32)	<0.001	0.06 (0.03–0.11)	<0.001	
Systolic blood pressure, per mmHg	1.00	1.01 (1.00–1.02)	0.204	1.01 (1.00–1.02)	0.167	0.083
Diastolic blood pressure, per mmHg	1.00	1.02 (1.00–1.04)	0.012	1.02 (1.01–1.04)	0.005	0.087
Triglycerides, per mmol/L	1.00	2.06 (1.41–3.02)	<0.001	2.70 (1.86–3.92)	<0.001	0.102
HDL-Cholesterol, per mmol/L	1.00	0.40 (0.24–0.66)	<0.001	0.27 (0.16–0.44)	<0.001	0.102
LDL-cholesterol, per mmol/L	1.00	1.15 (0.92–1.45)	<0.001	1.56 (1.26–1.93)	<0.001	0.092
Fasting plasma glucose, per mmol/L	1.00	0.93 (0.79–1.10)	0.417	1.18 (1.04–1.32)	0.007	0.090
HOMA-IR	1.00	1.42 (1.19–1.70)	<0.001	1.77 (1.49–2.10)	<0.001	0.183

*R*^2^ is the McFadden pseudo-*R*^2^ for the overall performance of the model containing age, sex, and the metabolic trait of interest.

## Abbreviations

The following abbreviations are used in this manuscript:

ANOVA	analysis of variance
ART	antiretroviral therapy
AIDS	acquired immunodeficiency syndrome
BMI	body mass index
B × M	interaction between BMI and metabolic status
BP	blood pressure
CVD	cardiovascular disease
DBP	diastolic blood pressure
FPG	fasting plasma glucose
HDL-C	high density lipoprotein cholesterol
HIV	human immunodeficiency virus
HOMA-IR	homeostatic model assessment of insulin resistance
Hs-CRP	high sensitivity-C reactive protein
LDL-C	low density lipoprotein cholesterol
M × G	interaction between metabolic status and gender
NWMH	normal-weight metabolically healthy
NWMA	normal weight metabolically abnormal
OvMH	overweight metabolically healthy
OvMA	overweight metabolically abnormal
OMH	obese metabolically healthy
OMA	obese metabolically abnormal
PDA	personal digital assistant
SBP	systolic blood pressure
SD	standard deviation
T2DM	type 2 diabetes mellitus
WC	waist circumference
